# High-throughput screening system for antimycobacterial compounds using *in vitro* infected cells: construction and application to compounds in the Ōmura Natural Compound Library

**DOI:** 10.1128/spectrum.00484-26

**Published:** 2026-06-30

**Authors:** Takemasa Takii, Aoi Kimishima, Masato Iwatsuki, Yoshihiro Watanabe, Naoya Ohara, Yukihiro Asami

**Affiliations:** 1 Department of Mycobacterium Reference and Research, The Research Institute of Tuberculosis, Japan Anti-Tuberculosis Association 543860, Kiyose, Tokyo, Japan; 2 Department of Hygienic Chemistry, Graduate School of Pharmaceutical Sciences, Nagoya City University 12963, Nagoya, Aichi, Japan; 3 Ōmura Satoshi Memorial Institute, Kitasato University 12877, Minato-ku, Tokyo, Japan; 4 Graduate School of Infection Control Sciences, Kitasato University 12877, Minato-ku, Tokyo, Japan; 5 Department of Oral Microbiology, Graduate School of Medicine, Density and Pharmaceutical Sciences, Okayama University 12997, Kita-ku, Okayama, Japan; Indian Institute of Science Bangalore, Bangalore, Karnataka, India

**Keywords:** *Mycobacterium tuberculosis*, *Mycobacterium avium*, *Mycobacterium abscessus*, fibroblasts, the Ōmura Natural Compound Library, antibacterial activity

## Abstract

The global increase in multidrug-resistant tuberculosis strains poses a major threat; consequently, there is an urgent need to identify and develop novel drugs. We have previously presented a simple fibroblast assay (SFA) screening method that enables the simultaneous assessment of antibacterial activity and cytotoxicity toward host cells. This method was developed based on our finding that live (but not dead) *Mycobacterium tuberculosis* bacilli exhibit cytotoxic activity against human lung fibroblasts, and that this cytotoxicity, which is proportional to the number of viable bacterial cells, is inhibited by known antimycobacterial drugs. While conventional methods to measure *M. tuberculosis* growth require 2–4 weeks to detect antibacterial activity; the SFA method enables detection within 2–3 days and can detect compounds that are active within host cells. Here, we applied high-throughput screening, based on assessment of cytotoxicity, to identify candidate antimycobacterial agents. In parallel, by comparing the direct antibacterial activity of compounds in liquid medium (using the broth dilution test) with that observed via SFA, we developed a new SFA-based screening system to select compounds with low cytotoxicity that are active outside or inside the host cells. Using this dual system, we screened 742 compounds from the Ōmura Natural Compound Library (Kitasato University, Japan), with 62 hits. Of these, 19 were excluded owing to their toxicity, and six exhibited antibacterial activity only via SFA screening. Four promising antimycobacterial candidates with no cytotoxicity at the 10 μg/mL threshold—aridicin A, lankamycin, streptovaricin, and lariatin A—were identified. This novel system supports the rapid identification of low-cytotoxicity antimycobacterial compounds that are active inside or outside the host cells.

**IMPORTANCE:**

This screening system, comprising the simple fibroblast assay (SFA) and broth dilution test (BDT), effectively identifies low-cytotoxicity compounds that exhibit antimycobacterial activity inside or outside the host cells.

## INTRODUCTION

Globally, approximately 1.7 billion people are infected with tuberculosis (TB), with 10.8 million cases and 1.25 million deaths (according to the World Health Organization), making it the most prevalent bacterial infectious disease and one of the top 10 causes of death (1). Multidrug-resistant (MDR) and rifampicin-resistant TB affect 410,000 people worldwide (1), and resistance to delamanid and bedaquiline, recently developed TB drugs, has already emerged. Without proper treatment, tuberculosis has a mortality rate of ca. 50%, and the increase in MDR strains poses a major threat (2).

The incidence of nontuberculous mycobacterial (NTM) disease, usually caused by infection with *Mycobacterium avium* complex (MAC) bacteria, such as *M. avium* and *Mycobacterium intracellulare*, is rapidly increasing. Furthermore, in recent years, the incidence of diseases caused by *Mycobacterium abscessus* complex (MABC) bacteria has increased (3). Both MAC- and MABC-associated diseases are difficult to treat, highlighting the urgent need to identify and develop novel drugs.

In antibiotic screening, the slow growth of acid-fast bacilli, such as *Mycobacterium tuberculosis*, presents a major challenge. *Mycobacterium tuberculosis* grows more slowly than bacteria, such as *Escherichia coli* and *Staphylococcus aureus*, taking several weeks to form colonies on agar media, and *M. avium*, the major pathogen causing NTM disease, requires approximately a week to form colonies. Therefore, using conventional methods that measure bacterial turbidity or colony formation, 3 to 4 weeks are required to screen for antibacterial agents against *M. tuberculosis*. Novel methods to rapidly determine the antibacterial activity of compounds against these bacilli are thus urgently required.

We have previously found that live (but not dead) *M. tuberculosis* bacterial cells induce cytotoxicity in human lung fibroblasts; this effect, which depends on the number of viable bacilli 2 days post-infection, declines as the number of bacilli is reduced by antibiotic treatment, and this suppression depends on the concentration of the antibiotic (4–6). Furthermore, using the Maestro Z live-cell analysis system (Axion BioSystems, Atlanta, GA), we have previously demonstrated that *M. tuberculosis*-induced fibroblast cell death occurs via pyroptosis (7). Based on the suppression of *M. tuberculosi*s-induced fibroblast death by anti-tuberculosis drugs, we developed the simple fibroblast assay (SFA) system to screen for compounds with anti-tuberculosis activity and assess their cytotoxicity (4, 5), and this method has been adopted in other studies (8, 9). Following infection with *Mycobacterium* bacilli, host cell death occurs within 2 days, and it is this rapid response to infection that enables the rapid measurement of antibacterial activity. The SFA method can also be used to measure the antimycobacterial activity of pyrazinamide, an anti-tuberculosis drug that is activated inside the host cells (6).

Here, to address the need for improved antibacterial screening methods, we developed a novel approach combining the SFA method with high-throughput screening of compounds in the Ōmura Natural Compound Library (Kitasato University (10–12). High-throughput screening was achieved using the recently developed Maestro Z live-cell analysis system (Axion BioSystems), which measures cytotoxic activity in real time (13).

## MATERIALS AND METHODS

### Antibiotics and natural compounds

The antibiotics, isoniazid, rifampicin, streptomycin, ethambutol, bedaquiline, and clarithromycin, were purchased from Sigma-Aldrich (St. Louis, MO). Rifampicin was used as the positive control at 0, 0.03, 0.06, 0.25, or 0.5 μg/mL. The natural compounds were provided by the Ōmura Satoshi Memorial Institute, Kitasato University, Minato-ku, Tokyo, Japan (10–12).

### Bacteria, culture, and frozen stock

*Mycobacterium tuberculosis* H37Rv and *Mycobacterium abscessus* were purchased from the American Type Culture Collection (#27294 and #19977, respectively; ATCC, Manassas, VA). *Mycobacterium avium* 104 was kindly provided by Dr. William Bishai of the Johns Hopkins School of Medicine, Baltimore. *Mycobacterium* bacilli were cultured in Middlebrook 7H9 broth (Difco, Detroit, MI) supplemented with 10% albumin dextrose catalase (ADC) (5% bovine serum albumin [fraction V], 2% dextrose, and 0.004% bovine liver catalase; Difco) and 0.05% Tween 80 and were cultured at 37°C under static conditions. Bacteria were grown to an optical density of 0.6 to 0.8 at 530 nm. The cultures were then aliquoted and stored at −80°C until use. The number of colony-forming units in each aliquot was determined via colony assay on Middlebrook 7H11 agar (Difco) supplemented with 10% oleic albumin dextrose catalase (0.05% oleic acid, 5% bovine serum albumin [fraction V], 2% dextrose, and 0.004% bovine liver catalase; Difco).

### Tissue cells and cell culture

We have previously applied SFA to the human embryonic lung fibroblast cell line MRC-5 (5, 6), a normal diploid cell line with limited cell division. Here, to obtain a stable host fibroblast line for SFA, we used MRC-5 SV1 TG1 cells (#RCB0207, JCRB, Tsukuba, Ibaraki, Japan), human embryonic lung fibroblast cells derived from the MRC-5 line and transformed with SV40 (14). MRC-5 SV1 TG1 cells are immortalized and therefore are easier to work with than MRC-5 cells, as there is no limit on subculturing, making them more suitable for high-throughput screening. Furthermore, as with the parental MRC-5 cells, MRC-5 SV1 TG1 cells are susceptible to anti-tuberculosis drugs. These cells were maintained in Dulbecco’s Modified Eagle Medium (low glucose) with L-glutamine, phenol red (Wako Pure Chemical Industries, Osaka, Japan), 100 μg/mL streptomycin (Meiji Seika Pharma, Tokyo, Japan), 100 units/mL penicillin G (Meiji Seika Pharma), and 5% heat-inactivated fetal bovine serum (Hyclone, GE Healthcare Life Sciences, Logan, UT). The culture was maintained at 37°C in 5% CO_2_ in a 100-mm diameter Falcon standard tissue-culture dish (No. 353003, Thermo Fisher Scientific, Waltham, MA).

### Measurement of cytotoxic activity of natural products using the SFA method

The SFA method was performed as previously described (5). MRC-5 SV1 TG1 cells were plated in 96-well flat-bottom microtiter wells, at 1.0 × 10^4^ cells per well in 100 μL of the culture medium, and then 100 μL of culture medium containing the natural compounds was added. After 3 days, the supernatant was removed. The cells were fixed in methanol for 1 min, stained with 0.75% crystal violet for 5 min, and rinsed with water. Wells in which the cells had detached from the plate and were not stained with crystal violet were visually identified as affected by cytotoxicity.

### Real-time measurement of cytotoxicity using Maestro Z

The Maestro Z system utilizes electrodes at the bottom of a 96-well plate to continuously measure the impedance of the adherent cells. Before seeding the fibroblasts onto 96-well plates (CytoView-Z, Axion BioSystems, Atlanta, GA) for use with the Maestro Z live-cell analysis system (Axion BioSystems, Atlanta, GA), the plates were coated with 100 μL of fibronectin (REF11051407001, Roche, Basel, Switzerland; 1 μg/mL) for 1 h at room temperature. Next, the coating solution was removed, and 100 μL of the tissue culture medium was added, followed by the addition of 8 mL of sterile water to the plate reservoirs to increase humidity. The plate was inserted into a Maestro Z chamber to measure the media-only baseline. The cells were diluted to 1.0 × 10^5^ cells/mL in DMEM containing 5% fetal bovine serum and 100 U/mL penicillin. Once the media-only baseline was obtained, the culture medium was removed from the plate, and 100 μL of the cell suspension was seeded on the plate. For tissue culture, the fibroblast-seeded plate was incubated in a Maestro Z chamber or a CO_2_ incubator.

### *In vitro* broth dilution method to measure minimum inhibitory concentration of antimycobacterial drugs and compounds

Broth dilution method to determine minimum inhibitory concentrations (MICs) of the antimycobacterial agents were prepared as described by Wallace et al. (15). Briefly, 100 μL of the drug solution was twofold serially diluted in Middlebrook 7H9 broth medium supplemented with 10% ADC and 0.05% Tween 80 and placed in 96-well plates, and 100 μL of broth medium containing 1 to 2 × 10^4^ organisms was added. The plates were then incubated at 37°C for 3 days for *M. abscessus*, for 1 week with *M. avium*, or 2 weeks for *M. tuberculosis*, after which turbidity was visually determined.

### Definition of MIC in SFA and BDT

In the SFA, MIC refers to the lowest concentration of the agent that significantly inhibits bacterial cytotoxicity, based on fibroblast viability (5). In the broth dilution test (BDT), in contrast, MIC refers to the lowest concentration of the agent that inhibits the visible growth of bacteria. We scored a partial reduction in turbidity as negative.

### Statistical analysis

The significance of effects was assessed via one-way ANOVA in SigmaPlot (Systat Software Inc., San Jose, CA).

## RESULTS

### Cytotoxicity of *M. tuberculosis* H37Rv and cytotoxicity and antibacterial activity of antimycobacterial agents

**Fig 1:**
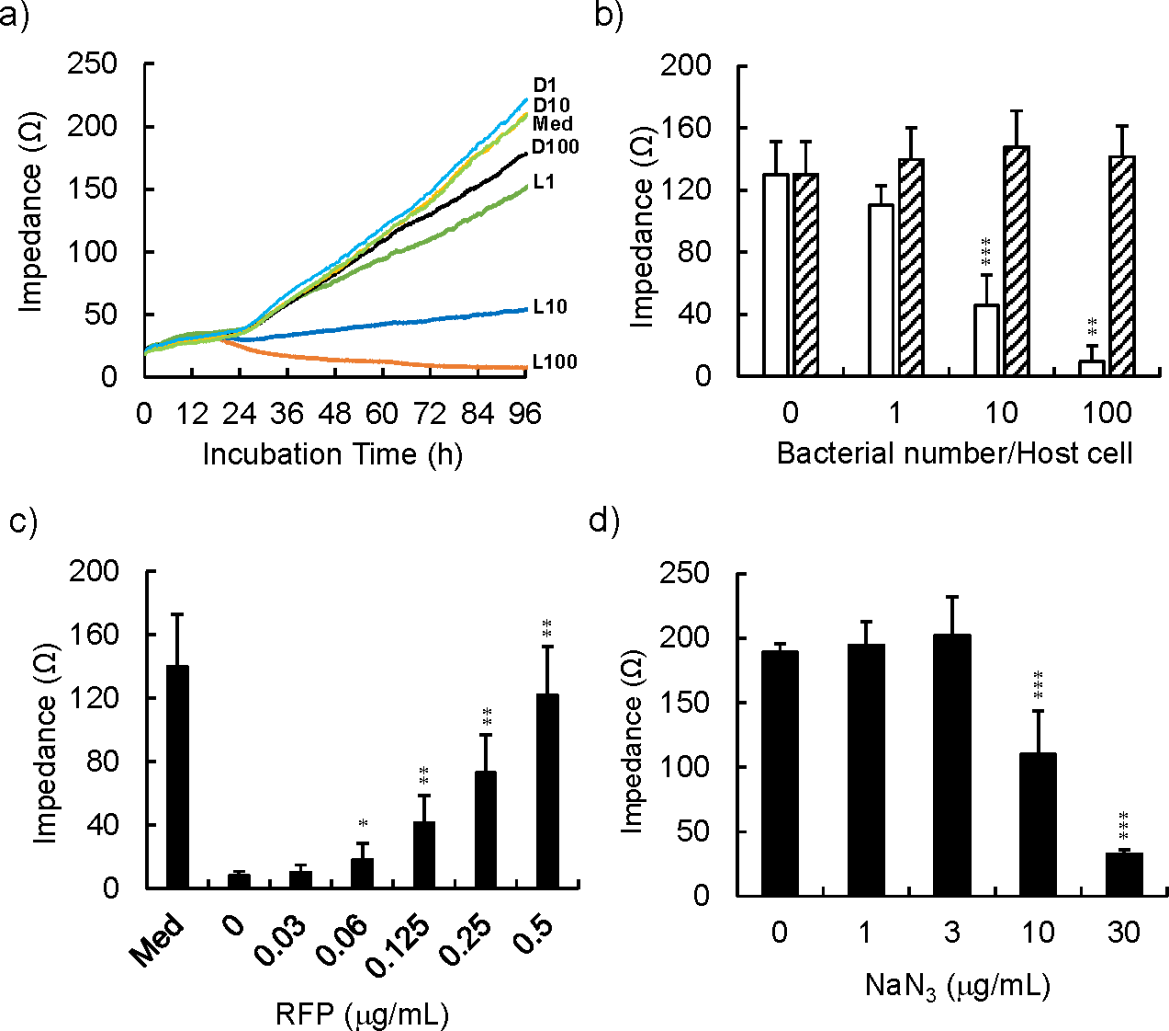
Cytotoxicity of *Mycobacterium tuberculosis* H37Rv and of natural compounds against MRC-5 SV1 TG1 host cells, based on real-time cytotoxicity assay using the Maestro Z live-cell analyzer (Axion BioSystems, Atlanta, GA). (**a**) Cytotoxic activity of *M. tuberculosis* H37Rv bacilli against the MRC-5 SV1 TG1 human lung fibroblast cell line. Horizontal axis: duration of coculture of MRC-5 SV1 TG1 cells and *M. tuberculosis* bacilli. The red (L100), dark blue (L10), and dark green (L1) lines represent live (“L”) *M. tuberculosis* bacilli at 100, 10, and 1 bacilli per host cell, while the black (D100), yellow (D10), light blue (D1) lines represent heat-killed (dead, “D”) bacilli at 100, 10, and 1 bacilli per host cell, respectively. The yellow-green lines represent the results with no bacteria (medium-only, “Med”). (**b**) Results after 72 h of the experiment shown in panel **a**. Shaded bars, dead bacteria; open bars, live bacteria. The experiments were replicated in six wells. Error bars indicate standard deviations. Statistical significance was assessed using one-way ANOVA. **P < 0.01, ***P < 0.001 vs no bacteria. (**c**) Inhibitory effects of rifampicin (RFP) on the cytotoxicity of *M. tuberculosis* H37Rv bacilli toward the host cells after 72 h. The MRC-5 SV1 TG1 cells (1 × 10^4^ cells/well) and bacilli (1 × 10^6^ bacilli per well) were cocultured for 72 h. The horizontal axis shows the wells without bacteria (Med) and the final concentration of RFP added 30 min before the bacilli. The experiments were replicated in six wells. Error bars indicate standard deviations. Statistical significance was assessed using one-way ANOVA. *P < 0.05, **P < 0.01 vs bacilli in the absence of RFP. (**d**) Cytotoxic activity of the energy uncoupler sodium azide (NaN_3_) against MRC-5 SV1 TG1 cells at 72 h. Experiments were replicated in six wells. Error bars indicate standard deviations. Statistical significance was assessed using a one-way ANOVA. ***P < 0.001 vs without sodium azide.

**Fig 2:**
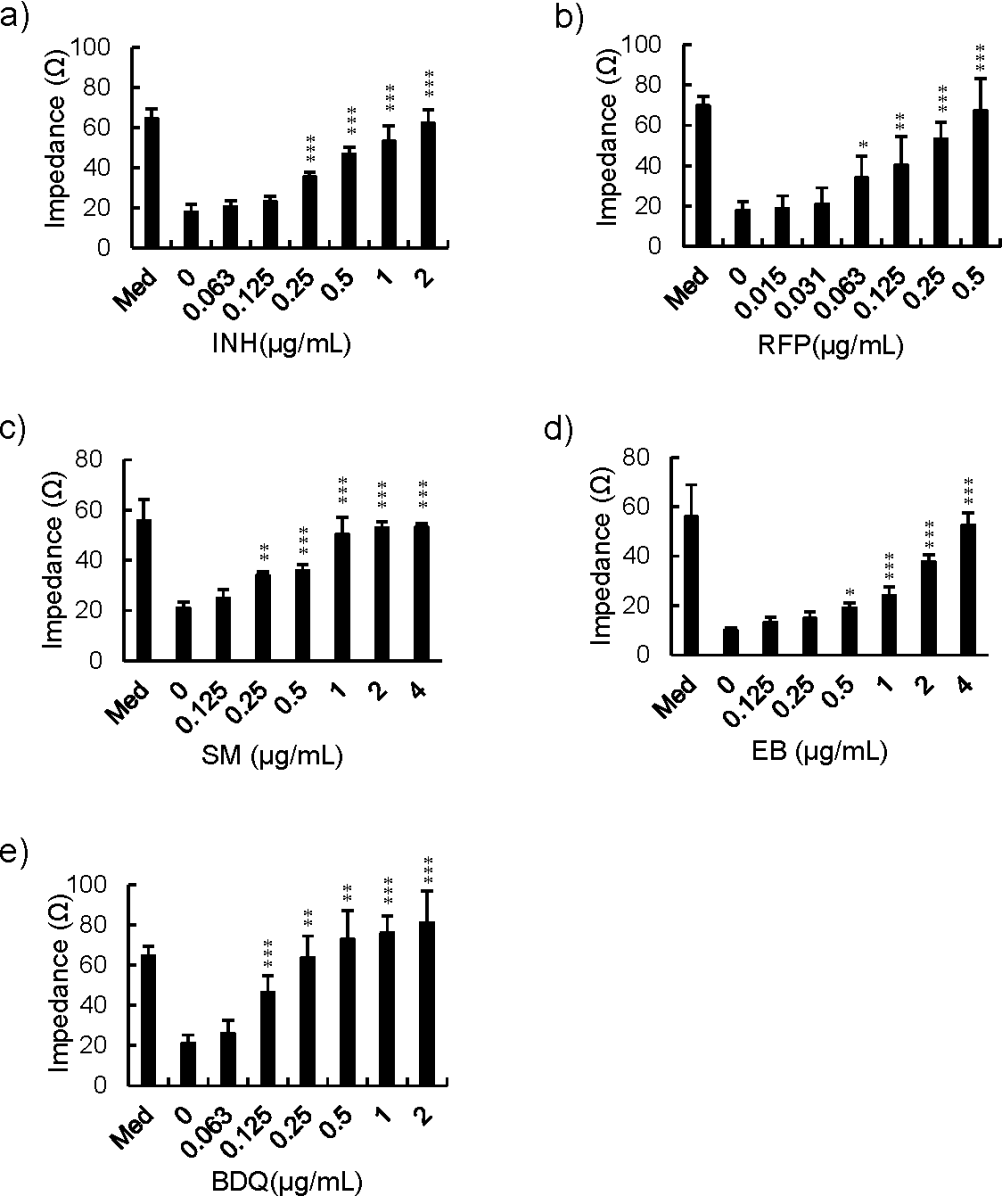
Anti-tuberculosis drug activity in MRC5 SV1 TG1 cells, based on the SFA method. MRC-5 SV1 TG1 cells were cultured for 3 days with live *M. tuberculosis* H37Rv bacilli in the presence of the antimycobacterial drugs (**a**) isoniazid (INH), (**b**) rifampicin (RFP), (**c**) streptomycin (SM), (**d**) ethambutol (EB), and (**e**) bedaquiline (BDQ) at the indicated concentrations. Cytotoxicity was measured using the Maestro Z real-time cytotoxicity assay system (Axion BioSystems, Atlanta, GA). Experiments were replicated in six wells. Error bars indicate standard deviations. Significance was tested by one-way ANOVA. *P < 0.05, **P < 0.01, ***P < 0.001, vs the bacteria without drugs.

As previously observed using MRC-5 cells (5, 6), host-cell death was induced in MRC-5 SV1 TG1 cells from 2 days after infection with live *M. tuberculosis* H37Rv bacilli in a bacterial number-dependent manner, whereas the presence of dead bacilli did not induce host-cell death (Fig. 1a and b). As in a prior study (5), we compared the MICs of anti-tuberculosis drugs, isoniazid, rifampicin, streptomycin, ethambutol, and betaquiline against *M. tuberculosis* H37Rv using the SFA method with those obtained using the liquid medium dilution test (BDT) method and obtained comparable results (Fig. 2; Table 1). The SFA method was effective for screening compounds (including sodium azide, an energy uncoupler) that are toxic to the MRC-5 SV1 TG1 host cells (Fig. 1d). We analyzed the antibacterial activity of the compounds on day 3 after infection, at which time the cytotoxic activity of *M. tuberculosis* H37Rv on the MRC-5 SV1 TG1 cells was stable (Fig. 1a and b).

**TABLE 1:** Comparison of MIC values^*a*^ using the SFA method and the BDT method

	INH	RFP	SM	EB	BDQ
BDT	0.125	0.03	0.25	1	0.25
SFA	0.25	0.06	0.25	0.5	0.125
SFA/BDT	2	2	1	1/2	1/2

^*a*^ MIC was obtained by SFA. MIC (μg/mL) determined by SFA is defined as the minimal dose of drugs exhibiting a statistically significant inhibitory effect on the bacterial cytotoxicity as examined by fibroblast viability (5). The values demonstrate statistically significant inhibition (*P* < 0.05) of the mycobacterial cytotoxicity. These assay procedures are described in the Materials and Methods. The values are means from six wells. The abbreviations for the antibiotics, isoniazid, rifampicin, streptomycin, ethambutol, and bedaquiline, are INH, RFP, SM, EB, and BDQ, respectively.

### Antimycobacterial activity of compounds in the Ōmura Natural Compound Library

We applied two stages of screening (Fig. 5) to identify candidates for antimycobacterial drug development in the Ōmura Natural Compound Library. In the first stage, the compounds’ antibacterial activity and cytotoxicity against the infected cells were evaluated via SFA. In parallel, the direct antibacterial activity of the compounds was evaluated via BDT, using a cutoff concentration for cytotoxicity of 10 μg/mL. Figure S1 presents an example of SFA measurements obtained using the Maestro Z device. Compounds exhibiting antibacterial activity equivalent to or greater than that of rifampicin at 0.25 μg/mL were considered as hit compounds.

### Compounds identified via primary screening

**Fig 3:**
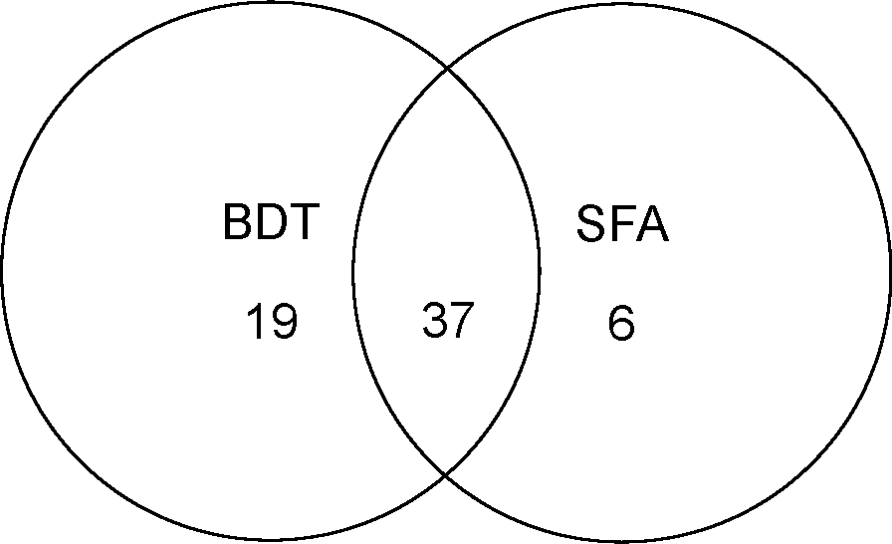
Venn diagram of the number of hits in the first stage of screening. The Venn diagram shows the respective proportions of the 62 hits obtained via the BDT and SFA methods.

During primary screening, *Mycobacterium avium*, the main causative bacterium of NTM disease, and *M. tuberculosis* were screened as the target bacterial species via BDT. From among the 742 compounds screened, primary screening via SFA and BDT identified 62 with antibacterial activity (Fig. 3; Table 2). Of these 62 compounds, 19 were cytotoxic to MRC-5 SV1 TG1 cells at a concentration of 10 μg/mL and were thus excluded from the list of candidates for antimycobacterial agents (Fig. 3; Table 3). Of the remaining candidates, 37 exhibited antibacterial activity without cytotoxicity (Fig. 3). Six of the candidates exhibited antibacterial activity only via SFA testing (Fig. 3; Table 2).

**TABLE 2:** Number of hit compounds in this screening method

Total number of compounds screened	742
First stage screening	
SFA (cutoff value^*a*^; 10 μg/mL)	43
Only SFA positive	6
BDT (cutoff value^*a*^; 10 μg/mL)	56
Only BDT positive (cytotoxic compound)	19
Second stage screening	
MIC (<1 µg/mL)^*b*^	8

^*a*^ Cutoff values for either *Mycobacterium tuberculosis* H37Rv ATCC 27294 or *Mycobacterium avium* 104.

^*b*^ MIC values (<1 µg/mL) for either *M. tuberculosis* H37Rv or *M. avium* 104.

**TABLE 3:** Minimum inhibitory concentration against *M. tuberculosis*, *M. avium*, and *M. abscessus^a^*

No.	Compound name	*M. tuberculosis* H37Rv ATCC 27294^*b*^	*M. avium* 104^*c*^	*M. abscessus* ATCC 19977^*d*^	Cytotoxicity^*e*^
Natural compounds					
1	Chlortetracycline	5	>10	>10	−
2	9-Propionyl maridomycin III	10	5	>10	−
3	Clindamycin	2.5	>10	>10	−
4	Aridicin A	0.31	10	2.5	−
5	Lividomycin B	1.25	10	10	−
6	Lincomycin	10	>10	>10	−
7	Lankamycin	<0.005	10	5	−
8	Methacycline	5	10	>10	−
9	Carbomycin B	>10	10	>10	−
10	Megalomycin A	>10	10	>10	−
11	Mycinamicin I	10	10	>10	−
12	Mycinamicin II	2.5	2.5	5	−
13	5-*O*-Mycaminosyl tylonolide	>10	10	>10	−
14	Ofloxacin	1.25	2.5	>10	−
15	Sagamicin	5	5	>10	−
16	Streptovaricin	0.04	1.25	>10	−
17	Ribostamycin	10	5	>10	+
18	Ostreogrycin A	10	10	>10	−
19	Streptomycin	0.31	2.5	5	−
20	Thiostrepton	5	10	5	−
21	Tylosin	>10	5	5	−
22	Tobramycin	10	5	>10	−
23	Tuberactinomycin N	2.5	>10	>10	−
24	Tunicamycin VII	0.31	0.08	>10	+
25	Tiamulin	10	10	>10	−
26	Erythromycin	>10	5	5	−
27	Hygromycin	1.25	10	>10	−
28	Quinomycin	0.04	0.63	0.63	+
29	Ristocetin A	0.31	>10	5	+
30	20-Bn_2_N Tylosin	1.25	5	>10	+
31	K41 Na	2.5	10	>10	+
32	Lariatin A	0.63	>10	5	−
33	Calcimycin	5	10	>10	+
34	Leucinostatin A	2.5	5	>10	+
35	Fusidic acid	5	10	>10	−
36	Chimeramycin A	>10	10	>10	−
37	Nanaomycin D	0.63	10	>10	+
38	Leucomycin A1	>10	10	>10	−
39	Leucomycin A3	>10	10	>10	−
40	Leucomycin A5	>10	10	>10	−
41	Dityromycin	10	>10	>10	−
42	Cerulenin	0.63	5	>10	+
43	Awamycin	0.16	5	>10	+
44	23-Deoxy-*O*-Mycaminosyltylonolide	10	>10	>10	−
45	Vineomycin A1	10	>10	>10	+
46	Cervinomycin	1.25	5	5	+
47	Pullularin B	10	>10	>10	−
48	Pullularin C	10	10	>10	−
49	Pullularin A	>10	10	>10	+
50	Questin	10	>10	>10	+
51	Ternatin	0.04	>10	>10	−
52	Borrelidin	0.16	10	>10	+
53	Beauvericin	5	10	>10	+
54	DC87 B	2.5	10	10	+
55	Xanthocillin X dimethyl ether	10	>10	>10	+
56	Rifamycin B	0.16	>10	>10	−
Antimycobacterial drug*^f^*					
	Rifampicin	0.06	0.06		
	Clarithromycin		0.25	0.063	

^*a*^ The MIC was determined by the liquid medium dilution method.

^*b*^ Antibacterial activity was assessed 2 weeks after the start of culture.

^*c*^ Antibacterial activity was assessed 1 week after the start of culture.

^*d*^ dAntibacterial activity was assessed on the third day of culture.

^*e*^ Cytotoxicity was assessed on the third day of culture using the SFA method with crystal violet staining. Detachment of cells from the 96-well plate was considered cytotoxic and was indicated by a "+" sign.

^*f*^ Antimicrobials used as positive control in the broth dilution method.

### Secondary screening to determine the MICs of the hit compounds

The MICs of the 37 compounds that tested positive via both BDT and SFA and of the 56 that tested positive via BDT were measured (Fig. 3 and 5; Table 2). The target bacterial species used for secondary screening were *M. tuberculosis* H37Rv, *M. avium* 104, and *M. abscessus* subsp. *abscessus*.

To examine the usefulness of the SFA method for simultaneously measuring antibacterial activity and cytotoxicity, we also measured the MICs of those compounds that were cytotoxic based on SFA (Table 3) but lacked antibacterial activity based on BDT.

**Fig 4:**
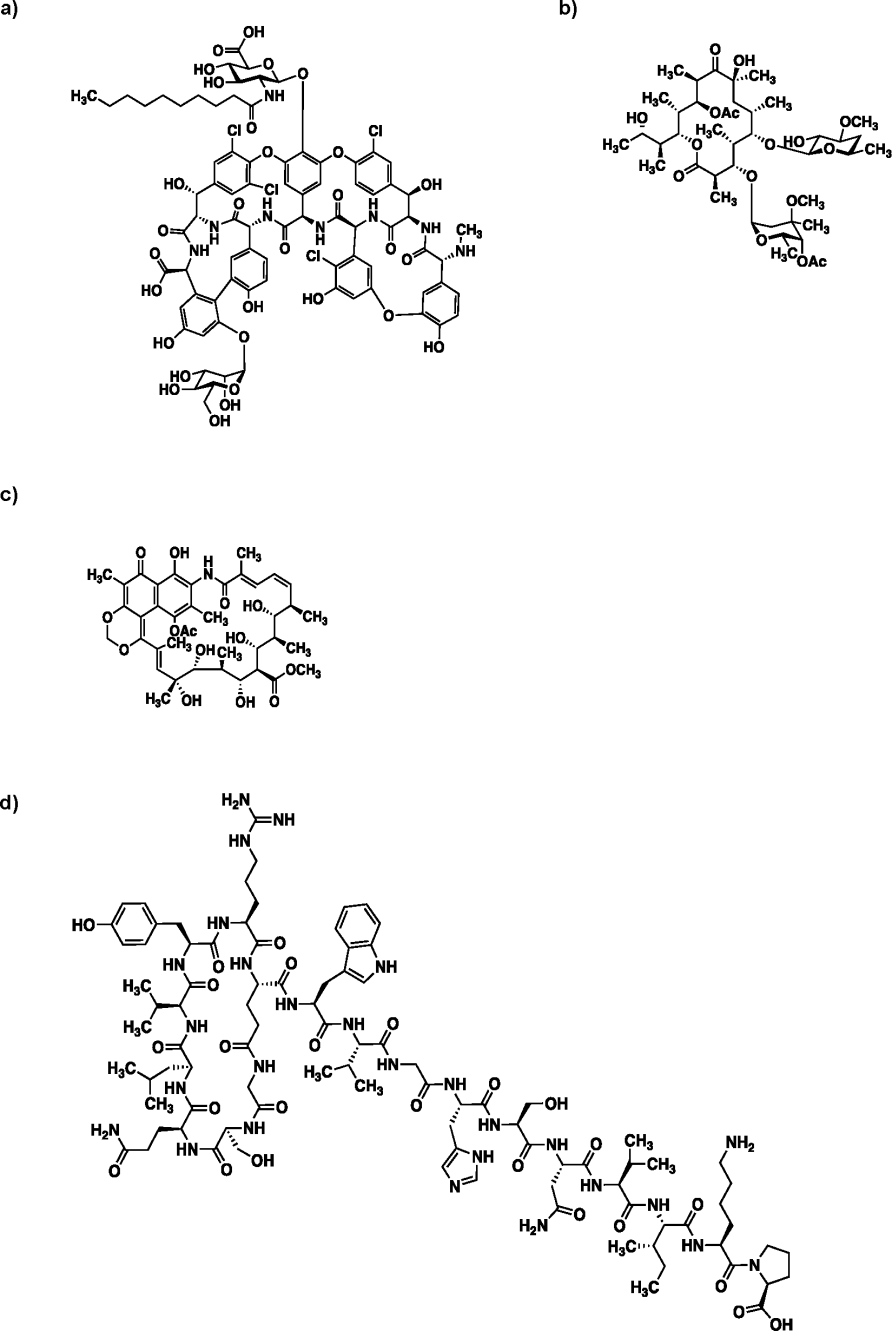
Structures of the four compounds selected from the Ōmura Natural Compound Library using our screening method as candidates for antimycobacterial drug development. Panels (**a–d**) show the structures of aridicin A, lankamycin, streptovaricin, and lariatin A, respectively.

Among the 62 compounds measured, 14 (ridicin A [no. 4], Lankamycin [no. 7], Streptovaricin [no. 16], Streptomycin [no. 19], Tunicamycin VII [no. 24], Quinomycin [no. 28], Ristocetin A [no. 29], Lariatin A [no. 32], Nanaomycin D [no. 37], Cerulenin [no. 42], Awamycin [no. 43], Ternatin [no. 51], Borrelidin [no. 52], and Rifamycin B [no. 56]) had MICs < 1 µg/mL against *M. tuberculosis* H37Rv (Fig. 4; Table 3). Of these, seven (Tunicamycin VII, Quinomycin, Ristocetin A, Nanaomycin D, Cerulenin, Awamycin, and Borrelidin) were cytotoxic and were therefore excluded from the list of candidates for anti-tuberculosis drug development. Of the remaining seven compounds, none showed MICs < 1 µg/mL against *M. avium* 104 or *M. abscessus* ATCC 19977. Among the seven that exhibited excellent MICs (MIC < 1 µg/mL) against *M. tuberculosis* H37Rv, two candidates—aridicin A and lankamycin—also showed antibacterial activity against *M. avium* 104 and *M. abscessus* ATCC 19977; both had MICs of 10 μg/mL against *M. avium* 104, while their MICs against *M. abscessus* ATCC 19977 were 2.5 and 5 μg/mL, respectively (Table 3). Aridicin A and lankamycin are therefore potential candidates for the development of antimycobacterial drugs.

Two other compounds, streptovaricin and lariatin A, showed excellent antibacterial activity against *M. tuberculosis* H37Rv, with MICs < 10 µg/mL against *M. avium* 104 or *M. abscessus* ATCC 19977. Streptovaricin had an MIC of 1.25 μg/mL against *M. avium* 104, but no antibacterial activity against *M. abscessus* (Table 3). In contrast, lariatin A had an MIC of 5 μg/mL against *M. abscessus* ATCC 19977, but no antibacterial activity against *M. avium* 104 (Table 3). Streptovaricin and lariatin A thus also represent potential candidates for developing antimycobacterial drugs.

## DISCUSSION

**Fig 5:**
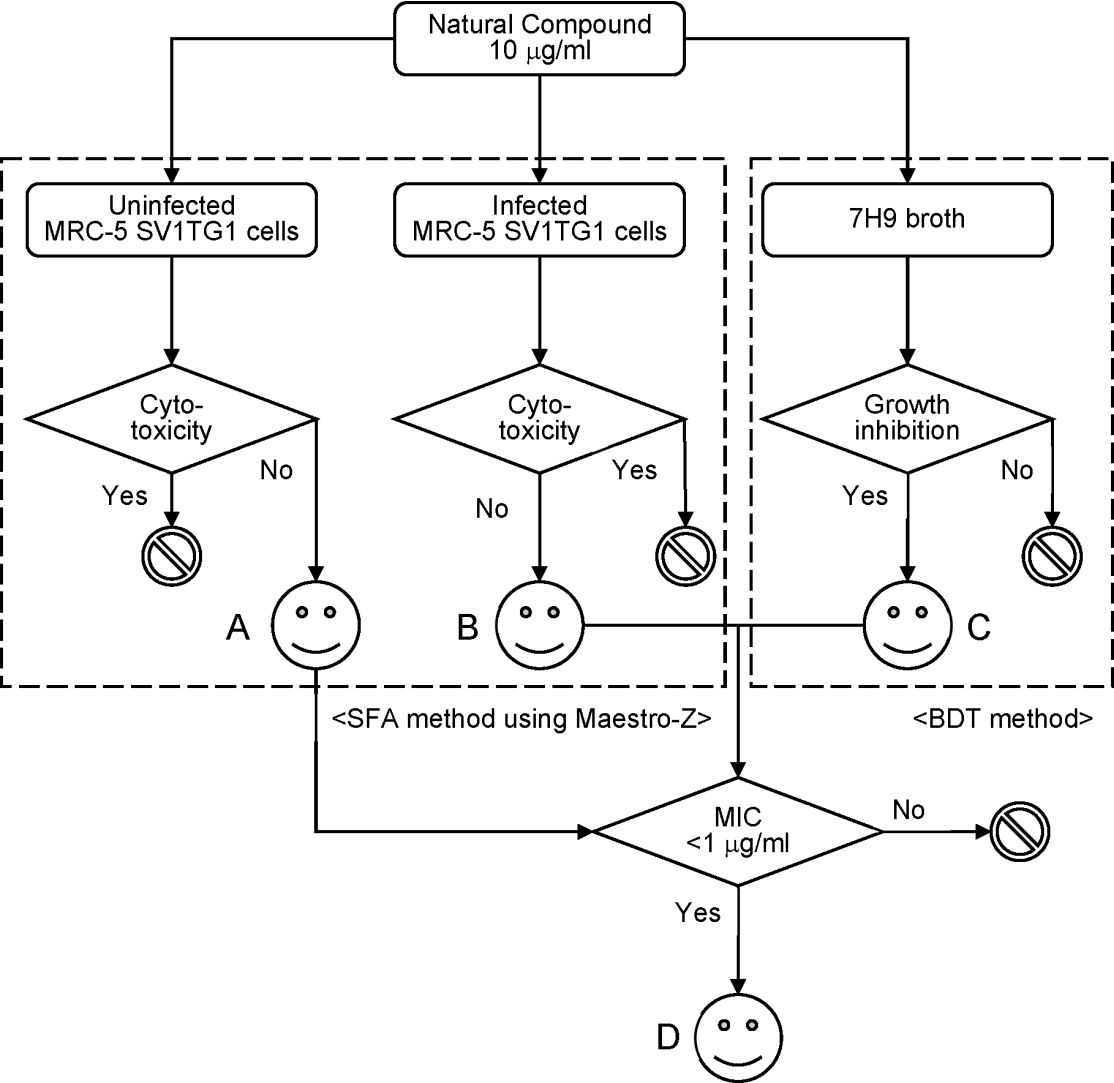
Flowchart of screening for compounds with antimycobacterial activity. In the first screening stage, the antibacterial activity and cytotoxicity of the natural products toward the host cells at 10 μg/mL are evaluated using the SFA method. Direct antibacterial activity is also evaluated using the BDT method. In the first stage, the hit compounds are selected based on criteria A–C. (Criterion A) Compounds with no cytotoxicity, determined using the SFA method; (criterion B) compounds that inhibit the cytotoxic activity of the bacteria in the infected host cells, determined using the SFA method (i.e., compounds that exhibit antibacterial activity against bacteria inside the host cells); and (criterion C) compounds that inhibit bacterial growth in liquid medium, determined using the BDT method. In the second stage, the MICs of the compounds selected in the first stage are measured, and those with MICs < 1 µg/mL are selected as candidate compounds (criterion D) for the development of antimycobacterial drugs.

This study presents a novel high-throughput screening system, based on the SFA and BDT methods and utilizing the Maestro Z live-cell analyzer, for identifying anti-tuberculosis candidate drugs (Fig. 5). Consistent with our prior findings (4, 5), *M. tuberculosis*-induced host cell death occurred from day 2 (Fig. 1a and b). For the antimicrobial activity of INH, RFP, SM, and EB against *M. tuberculosis* bacilli, the MIC values measured by the BDT method were used as the gold standard. The ratio of MIC values measured by the SFA method and the BDT method, SFA/BDT, was compared with our prior study (5). In our prior study using MRC-5, the ratios were 4, 1/2, 8, and 2, respectively (5). In this study, we improved upon our prior study by changing the host cell to MRC-5 SV1 TV1 and using Maestro Z, resulting in ratios of 2, 2, 1, and 1/2, respectively (Table 1).

Our aim in first-pass screening was to evaluate, at one concentration and under the same conditions, both the intracellular activity/cytotoxicity (via SFA) and direct extracellular activity (via BDT). The cytotoxic-concentration threshold applied here (10 μg/mL) was chosen a priori as an operational threshold for primary screening, and not as a statistically optimized pharmacological cutoff. We selected this threshold as a compromise concentration that is high enough to avoid missing moderately active compounds (including those preferentially detectable in the host cell-based SFA format) and low enough to exclude compounds that are clearly inactive or notably cytotoxic. Our primary screening using this threshold yielded a manageable hit set (62 hits out of 742 compounds) (Fig. 3; Tables 2 and 3), while excluding 19 cytotoxic compounds.

This excluded cytotoxic compounds such as sodium azide (Fig. 1d), consistent with our prior findings (5). Secondary screening, based on MIC determination, was then used to apply a more stringent threshold, with compounds showing an MIC < 1 µg/mL considered development candidates. Secondary screening identified multiple potential antimycobacterial candidates, although some of these exhibited cytotoxicity.

Tunicamycin (no. 24) exhibits antibacterial activity against *Bacillus* (MIC 0.1–20 μg/mL) (16), but also exhibits cytotoxicity against eukaryotic cells by inhibiting eukaryotic N-linked glycoprotein biosynthesis (17). Owing to its characteristic mode of action, tunicamycin has attracted attention as a potential lead in the development of new drug candidates against TB and NTM infections (18). It has been studied for its potential use as an anticancer drug because it induces apoptosis via endoplasmic reticulum stress (19). In human prostate cancer PC-3 cells, tunicamycin caused a dose-dependent reduction in cell viability at concentrations of 1–10 µg/mL, with 10 µg/mL being close to the median toxic dose (TD_50_) after 72 h of treatment (20). Here, tunicamycin exhibited excellent antibacterial activity against *M. tuberculosis* H37Rv and *M. avium* 104, with MICs of 0.31 and 0.08 μg/mL, respectively, although it exhibited cytotoxicity against MRC-5 SV1 TG1 cells at 10 μg/mL. It was thus excluded owing to its cytotoxicity.

Quinomycin (echinomycin, no. 28) showed excellent antibacterial activity against *M. tuberculosis* H37Rv, *M. avium* 104, and *M. abscessus* ATCC 19977 bacilli, with MICs of 0.04, 0.63, and 0.63 μg/mL, respectively, but was cytotoxic (Table 3). Quinomycin, which acts as an intercalator that binds to DNA, exhibits excellent antibacterial activity against pathogenic bacteria, such as *Streptococcus pneumoniae*, *Staphylococcus aureus*, *Listeria monocytogenes*, and *Enterococcus faecalis*, with MICs of <0.03, 0.25–1, 0.25–0.5, and 0.13 μg/mL, respectively (21). It exhibits moderate antibacterial activity against TB and NTM, both *in vitro* and in a silkworm infection model (22, 23). Quinomycin actively inhibits hypoxia-inducible factor HIF-1 in human cells and is therefore under study as an anticancer drug (24). However, as it exhibits cytotoxicity against human mammary epithelial cells, a primary human cell line, with an IC_50_ of 4.8 ± 1.1 µg/mL (21), it was excluded from the list of candidates for further development.

Ristocetin A (no. 29), a natural glycopeptide like vancomycin, exhibited excellent antibacterial activity against *M. tuberculosis* H37Rv ATCC 27294 and *M. abscessus* ATCC 19977, with MICs of 0.31 and 5 μg/mL, respectively (Table 3). Nonetheless, its antibacterial activity against TB and NTM is generally limited (25). Although it has been used to treat staphylococcal infections, it is no longer used clinically owing to its side effects (thrombocytopenia and agglutination) (26). Ristocetin A is now used only to diagnose von Willebrand disease and Bernard-Soulier syndrome and is therefore unsuitable for treating mycobacterial diseases that require long-term administration (26). Ristocetin A was therefore excluded from the list of candidates for further investigation.

Cerulenin (no. 42), an antifungal antibiotic and the first natural antibiotic known to inhibit fatty acid and steroid biosynthesis (27), showed excellent antibacterial activity against *M. tuberculosis* H37Rv and *M. avium* 104, with MICs of 0.63 and 5 μg/mL, respectively (Table 3). Cerulenin exhibits antibacterial and antifungal activities against both bacteria and fungi, including *Staphylococcus aureus* FDA 209P, *Mycobacterium smegmatis* (ATCC 607), *E. coli* NIHJ, *Pseudomonas aeruginosa* P-3, *Candida albicans*, and T*richophyton interdigitale,* with MICs of 100, 1.56, 12.5, >100, 1.56, and 12.5 μg/mL, respectively (28). It exhibits MICs of 1.5–12.5 μg/mL against *M. tuberculosis* H37Rv, clinical isolates of *M. tuberculosis*, the *M. avium-intracellulare* complex, *Mycobacterium kansasii*, and *M. smegmatis* (29). Cerulenin exhibits selective toxicity in both cancer and normal cells. For instance, it exhibits cytotoxicity against multiple melanoma cell lines and MCF-7 human breast cancer cells, with IC_50_ values of 2.0–4.9 μg/mL (48 h) and 5 μg/mL (24 h), respectively (30, 31). At 22 μg/mL, it reduced the viability of peripheral blood mononuclear cells and plasma cells from healthy volunteers by 20% within 24 h (30). In mice, an intraperitoneal dose of cerulenin at 30 mg/kg inhibited feeding and induced significant weight loss (32). Cerulenin was therefore excluded owing to its high toxicity.

Awamycin (no. 43), an ansamycin antibiotic, exhibits excellent antibacterial activity against *Staphylococcus aureus* FDA 209P, *Bacillus subtilis* PCI 219, *Bacillus cereus* IFO 3001, and *Micrococcus luteus* PCI 1001, with MICs of 0.1, 0.8, 0.8, and 0.05 μg/mL, respectively (33). It inhibits the proliferation of HeLa human cervical cancer cells at 2.5 μg/mL (33). Here, awamycin exhibited excellent antibacterial activity against *M. tuberculosis* H37Rv and *M. avium* 104, with MICs of 0.16 and 5 μg/mL, respectively (Table 3), a finding not previously reported. Nonetheless, it exhibited cytotoxicity at 10 μg/mL and was therefore excluded from the list of candidates.

Nanaomycin D (no. 37, MIC 0.63 μg/mL) and borrelidin (no. 52, MIC 0.16 μg/mL) exhibited excellent antibacterial activity against *M. tuberculosis* H37Rv ATCC 27294 with MICs < 1 µg/mL, but lacked activity against *M. avium* 104 or *M. abscessus* ATCC 19977 (with MICs of 10 μg/mL) (Table 3). However, both were excluded as candidates based on their cytotoxicity at 10 μg/mL (Table 3).

Among the compounds that showed no cytotoxicity at 10 μg/mL (Fig. 5, lane 1), but showed antibacterial activity at 10 μg/mL (Fig. 5, lanes 2 and 3), four compounds—aridicin A, lankamycin, streptovaricin, and lariatin A—showed excellent antibacterial activity against *M. tuberculosis* H37Rv, *M. avium* 104, and *M. abscessus* ATCC 19977, with MICs < 1 µg/mL (Table 3), thus representing promising antimycobacterial drug candidates (Fig. 4).

Aridicin A (no. 4), a glycopeptide antibiotic like aristocetin A and vancomycin, exhibits excellent antibacterial activity against *Staphylococcus aureus* 127, *Streptococcus faecalis* 34358, *L. monocytogenes* 2255, and *Clostridioides difficile*, with MICs of 1.6, 0.8, 0.8, and 0.13 μg/mL, respectively (34). However, its antimycobacterial activity remains unknown. Here, aridicin A showed excellent antibacterial activity against *M. tuberculosis* H37Rv ATCC 27294 and *M. abscessus* ATCC 19977, with MICs of 0.31 and 2.5 μg/mL, respectively (Table 3). Considering its structural similarity to the other glycopeptide antibiotics, aridicin A is expected to possess functional groups conducive to its antibacterial activity against *M. tuberculosis* and *M. abscessus*. This is the focus of our subsequent investigation. Aridicin A has been found to exhibit comparable antibacterial activity to vancomycin against *Staphylococcus aureus* HH127 MS, *Staphylococcus aureus* 2620 MR, *Staphylococcus epidermidis* 2479 MS, *Streptococcus faecalis* HH34358, *Streptococcus faecalis* 657, and *Streptococcus pyogenes* C203, with MICs of 3.1, 6.3, 25, 0.8, 0.8, and 0.2 μg/mL, respectively; further, in mice infected with these bacilli, it exhibits comparable efficacy to vancomycin (35). In that mouse model, subcutaneous injection with aridicin A achieved a higher peak blood concentration and longer blood half-life than subcutaneous injection with vancomycin (35).

Lankamycin (Kujimycin B, no. 7), a macrolide antibiotic, exhibits moderate antibacterial activity against several *Staphylococcus* strains, with MICs of 3–12 μg/mL (36). Here, lankamycin showed excellent antibacterial activity against *M. tuberculosis* H37Rv ATCC 27294 and *M. abscessus* ATCC 19977, with MICs of <0.005 and <5 µg/mL, respectively (Table 3). While most of the 16-membered macrolides exhibit limited activity against *M. tuberculosis* and *M. abscessus*, lankamycin exhibits significantly better activity, thus underscoring its potential for further development (37).

Streptovaricin (no. 16), an ansamycin antibiotic structurally highly similar to the rifamycins, has been found to inhibit *M. tuberculosis* growth *in vitro* and to exert chemotherapeutic effects in a mouse model of *M. kansasii* infection (38). Here, streptovaricin exhibited excellent antibacterial activity against *M. tuberculosis* H37Rv and *M. avium* 104, with MICs of 0.04 and 1.25 μg/mL, respectively (Table 3). It exhibits moderate antibacterial activity against *Staphylococcus aureus* (multiple strains), *M. tuberculosis* H37Rv, and *Mycobacterium bovis* BCG, with MICs of 0.78–3.13, 1.56, and 1.56 μg/mL, respectively (39).

Lariatin A (no. 7), a lasso peptide produced by *Rhodococcus jostii*, exhibits antibacterial activity against *M. smegmatis*, with an MIC of 0.1 μg/mL (40, 41). In other studies, it has exhibited excellent antibacterial activity against *M. smegmatis*, *M. avium* JCM 15430, *M. intracellulare* JCM 6384, *M. bovis* BCG Pasteur, and *M. abscessus* ATCC 19977, with MICs of 0.1, 0.78, 1.56, 0.78, and 1.56 μg/mL (22, 42). In silkworm models of *M. smegmatis* and *M. abscessus* infection, lariatin A exhibited ED_50_ values of 0.5 μg/g larva and 8.84 μg/g larva, respectively, performing similarly to existing anti-tuberculosis drugs (22, 42). Here, lariatin A showed excellent antibacterial activity against *M. tuberculosis* H37Rv ATCC 27294 and *M. abscessus* ATCC 19977, with MICs of 0.63 and 5 μg/mL, respectively (Table 3).

Our screening system identified various compounds (Table 3) with previously reported mycobacterial activity, namely streptovaricin (39), lariatin A (22, 42), quinomycin (22), cerulenin (28), ofloxacin (43), streptomycin (44), tobramycin (45), erythromycin (46), and hygromycin (47), thus demonstrating the rapidity and reliability of this method. Although we were blinded to the identity of each compound during screening, we specifically selected compounds with reported antimycobacterial activity for screening.

In this study, although the names of the compounds were kept confidential during screening, we were able to select compounds with reported antimycobacterial activities as described above. The fact that the SFA method eliminated compounds with cytotoxic activity against human fibroblasts at the beginning of the screening is extremely useful for developing antibacterial drugs. Four compounds, aridicin A, lankamycin (kujimycin B), streptovaricin, and lariatin A, which showed excellent antibacterial activity against *M. tuberculosis* H37Rv ATCC 27294, *M. avium* 104, and *M. abscessus* ATCC 19977 bacilli in the second stage screening, were identified as promising new antimycobacterial drug candidates (Fig. 4).

The SFA-only hits were retained as potentially host-cell-active compounds and are important subjects for future mechanistic study (Fig. 3; Table 2).

### Conclusions

The SFA method eliminated compounds that are cytotoxic toward human fibroblasts in the primary screening stage, thus highlighting its usefulness in the development of antibacterial drugs. Integrating a real-time cytotoxicity assay into the SFA method enabled rapid screening of multiple samples, and combining it with the BDT method enabled efficient screening and elimination of cytotoxic compounds. Finally, the six compounds identified here that are active only within the infected cells represent antibacterial agents with novel mechanisms of action.

## SUPPLEMENTAL MATERIAL

Figure S1 spectrum.00484-26-s0001.tiff Example of the measurement of anti-tuberculosis activity of compounds in the Omura Natural Compound Library using the Maestro Z system.

ASM does not own the copyrights to Supplemental Material that may be linked to, or accessed through, an article. The authors have granted ASM a non-exclusive, world-wide license to publish the Supplemental Material files. Please contact the corresponding author directly for reuse.
